# Stereo Viewing Modulates Three-Dimensional Shape Processing During Object Recognition: A High-Density ERP Study

**DOI:** 10.1037/xhp0000444

**Published:** 2017-10-12

**Authors:** Zoe J. Oliver, Filipe Cristino, Mark V. Roberts, Alan J. Pegna, E. Charles Leek

**Affiliations:** 1 School of Psychology, Bangor University; 2 School of Psychology, University of Queensland; 3 School of Psychology, Bangor University

**Keywords:** 3D object recognition, evoked potentials, local and global shape, stereo disparity

## Abstract

The role of stereo disparity in the recognition of 3-dimensional (3D) object shape remains an unresolved issue for theoretical models of the human visual system. We examined this issue using high-density (128 channel) recordings of event-related potentials (ERPs). A recognition memory task was used in which observers were trained to recognize a subset of complex, multipart, 3D novel objects under conditions of either (bi-) monocular or stereo viewing. In a subsequent test phase they discriminated previously trained targets from untrained distractor objects that shared either local parts, 3D spatial configuration, or neither dimension, across both previously seen and novel viewpoints. The behavioral data showed a stereo advantage for target recognition at untrained viewpoints. ERPs showed early differential amplitude modulations to shape similarity defined by local part structure and global 3D spatial configuration. This occurred initially during an N1 component around 145–190 ms poststimulus onset, and then subsequently during an N2/P3 component around 260–385 ms poststimulus onset. For mono viewing, amplitude modulation during the N1 was greatest between targets and distracters with different local parts for trained views only. For stereo viewing, amplitude modulation during the N2/P3 was greatest between targets and distracters with different global 3D spatial configurations and generalized across trained and untrained views. The results show that image classification is modulated by stereo information about the local part, and global 3D spatial configuration of object shape. The findings challenge current theoretical models that do not attribute functional significance to stereo input during the computation of 3D object shape.

## Public Significance Statement

The aim of this research is to elucidate how the human visual system processes sensory information about shapes of 3-dimensional (3D) objects so that we can perceive, and recognize, them. We asked whether these processes are sensitive to both monocular and stereo visual input. To answer this question we measured electrophysiological responses generated in the brain while people viewed, and made recognition judgments about, mono or stereo images of 3D objects. The objects could differ from each in terms of their part structure, or overall 3D spatial configuration. The results showed that the visual system processes these sorts of shape properties differently, and that how it does so is influenced differently by mono and stereo visual input. The findings shed new light on the role of stereo information in the visual perception and recognition of 3D object shape.

The human visual system is remarkable for its ability to rapidly and accurately classify three-dimensional (3D) objects despite variability in sensory input (e.g., Arguin & Leek, 2003; Bar, 2003; Bar, Kassam, Ghuman, et al., 2006; Cichy, Pantazis, & Oliva, 2014; Fabre-Thorpe, 2011; Harris, Dux, Benito, & Leek, 2008; Kirchner & Thorpe, 2006; Leek, 1998a, 1998b; Leek, Atherton, & Thierry, 2007; Leek, Davitt, & Cristino, 2015; Leek & Johnston, 2006; Leek, Roberts, Oliver, Cristino, & Pegna, 2016; Lloyd-Jones, Roberts, Leek, Fouquet, & Truchanowicz, 2012; Tarr & Bulthoff, 1998; Thorpe, Fize, & Marlot, 1996; VanRullen & Thorpe, 2001).

One important, and unresolved, issue is whether, and under what conditions, information derived from stereo (binocular) disparity influences the recognition of 3D object shape (e.g., Bennett & Vuong, 2006; Chan, Stevenson, Li, & Pizlo, 2006; Cristino, Davitt, Hayward, & Leek, 2015; Edelman & Bülthoff, 1990; Koenderink, van Doorn, & Kappers, 1992; Li, Pizlo, & Steinman, 2009; Norman, Todd, & Phillips, 1995; Pegna, Darque, Roberts, & Leek, 2016; Pizlo, Sawada, Li, Kropatsch, & Steinman, 2010; Welchman, Deubelius, Conrad, Bul̆thoff & Kourtzi, 2005). Some current theories attribute little, if any, significance to stereo information (e.g., Bülthoff & Edelman, 1992; Chan et al., 2006; Pizlo, 2008; Riesenhuber & Poggio, 1999; Serre, Oliva, & Poggio, 2007). For example, in the HMAX model (Riesenhuber & Poggio, 1999), image classification is accomplished within a multilayer feedforward architecture in which hierarchically structured edge-based representations of object shape are computed from monocular image contour—see also other recent approaches to image classification based on hierarchical deep networks (e.g., Cichy, Khosla, Pantazis, Torralba, & Oliva, 2016; Khaligh-Razavi & Kriegeskorte, 2014; Krizhevsky, Sutskever, & Hinton, 2012). Pizlo (2008; see also Li et al., 2009; Pizlo et al., 2010) has proposed that 3D object structure is computed solely from 2D shape information subject to the application of simplicity constraints (symmetry, compactness, planarity and minimum surface area). On other accounts, the contribution of stereo input is not ruled out, but neither explicitly incorporated into the proposed theoretical framework (e.g., Biederman, 1987; Leek, Reppa, & Arguin, 2005; Ullman, 2007). This contrasts with theoretical models that have attributed functional significance to certain kinds of stereo-defined shape information in object recognition—such as the computation of local surface depth orientation, and the specification of 3D object structural descriptions (Marr & Nishihara, 1978).

Although binocular disparity has been shown to contribute to the perception of surface properties such as slant, tilt, and curvature (e.g., Ban & Welchman, 2015; Norman et al., 1995; Norman et al., 2009; Welchman et al., 2005; Wexler & Ouarti, 2008; Wismeijer, Erkelens, van Ee, & Wexler, 2010), its role in the recognition of complex 3D object shape remains unclear. Indeed, it has been argued that although stereo information (i.e., local depth disparity) facilitates processing of 3D surfaces properties this does not, in itself, establish a functional link between stereo vision and the perception (and recognition) of complex (i.e., multipart) 3D object shape per se (Li et al., 2009; Pizlo, 2008; Pizlo et al., 2010). This issue has been investigated in previous studies by assessing the effects of stereo disparity on the perceptual matching of object shape across changes in viewpoint. The results provide a mixed picture with stereo advantages reported in some studies (e.g., Bennett & Vuong, 2006; Burke, 2005; Burke, Taubert, & Higman, 2007; Chan et al., 2006; Edelman & Bülthoff, 1990; Hong Liu, Ward, & Young, 2006; Lee & Saunders, 2011; Rock & DiVita, 1987; Simons, Wang, & Roddenberry, 2002), but not in others (Humphrey & Khan, 1992; Pasqualotto & Hayward, 2009). Recently, Cristino et al. (2015) have proposed that stereo information is computed during the visual perception of object shape. It is more likely to be used to supplement shape information derived from mono-ocular cues when object recognition (i.e., target/nontarget discrimination or view generalization) is facilitated by the derivation of 3D object structure. In support of this hypothesis, they showed that stereo input facilitates the classification of complex multipart 3D objects across large, but not small, changes in depth rotation. In other recent work, Pegna et al. (2016) have found early perceptual sensitivity to stereo versus mono input in a perceptual matching task using event-related potentials (ERPs). In that study, ERPs were recorded while observers made shape equivalence judgments about pairs of sequentially presented novel 3D objects under conditions of stereo or mono viewing. The results showed an early perceptual sensitivity to the mode of input shown by a negative amplitude modulation between 160 and 220 ms poststimulus onset. The results also showed later modulation of ERP amplitude during an N2 component between 240 and 370 ms for stereo and mono input that was linked to the perceptual matching of shape.^1^

The aim of the current study was to determine whether stereo disparity contributes to object processing during the recognition of 3D object shape. The rationale was based on recent work by Leek et al. (2016), who found evidence for early differential sensitivity of ERP amplitudes to local part structure and global shape configuration of complex 3D objects in mono displays. In that study ERPs were recorded while observers made shape matching judgments to sequentially presented pairs of novel objects under conditions of mono viewing. Different object pairs could either share local parts but differ in global shape configuration, share global shape configuration but have different local parts, or share neither. The results showed differential N1 sensitivity to local and global shape similarity between stimulus pairs occurring around 170 ms poststimulus onset. These findings provide evidence that mental representations of complex 3D object shapes comprise both local higher-order parts, and the global spatial configuration of these parts—consistent with theoretical models, and other empirical evidence, supporting this distinction (e.g., Arguin & Saumier, 2004; Behrmann, Peterson, Moscovitch, & Suzuki, 2006; Behrmann & Kimchi, 2003; Biederman, 1987; Hummel, 2013; Hummel & Stankiewicz, 1996; Marr & Nishihara, 1978). We hypothesized that one way in which stereo disparity may contribute to recognition is by facilitating the computation of 3D object representations via depth information. These representations could augment a range of shape information including surface depth gradients and curvature, higher-order part boundaries, and the 3D spatial configuration of (volumetric) object parts. Of relevance to the current study is whether stereo input might differentially modulate the sensitivity of object recognition processes to local part and global 3D spatial configuration information. For example, under some structural description accounts, object parts are computed directly from 2D image-based input derived from local edge relations (e.g., nonaccidental properties or NAPs—Biederman, 1987). This level of representation may be sufficient where object recognition can be based on a parts-based description of object identity, or where the discrimination of target and nontarget objects can be achieved based on part composition. In other situations, it may be beneficial to compute a global 3D object model which specifies (among other attributes) the spatial configuration of local object parts—for example, where recognition depends on discrimination among objects with similar parts but different spatial configurations.

To test this prediction we used ERPs, which have been previously shown by Leek et al. (2016) to show differential amplitude sensitivity to local and global shape structure. Unlike earlier work, we also wanted to examine this issue in the context of an object recognition task rather than the perceptual matching of sequentially presented stimuli. Object recognition differs from perceptual matching in that the former requires indexing a (stored) long-term memory representation of object shape. We used a recognition memory task in which observers had to first memorize a subset of complex novel 3D objects (targets) and subsequently discriminate them from visually similar nontarget (not previously memorized) objects. We then contrasted effects of target/nontarget similarity defined by local part and global 3D shape configuration under conditions of stereo and mono viewing. We predicted that stereo presentation would enhance ERP modulations related to object discrimination weighted toward perceptual analysis of 3D global shape configuration.

## Method

### Participants

Forty Bangor University students (24 female, mean age 21.46, *SD* = 3.16, 3 left-handed) participated for course credit. The sample was recruited through an online participation portal. All participants had normal or corrected-to-normal visual acuity. Ethics approval was granted by the Local Ethics Committee and in accordance with British Psychological Society guidelines. Informed consent was obtained and participants were free to withdraw from the study at any time without penalty.

### Apparatus and Stimuli

**Figure 1 fig1:**
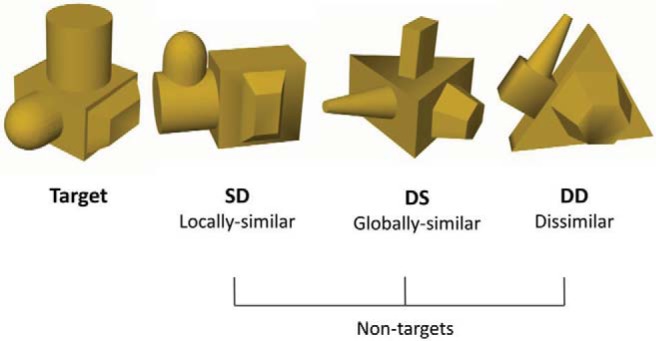
An example of one target object and its three corresponding SD (locally similar), DS (globally similar), and DD (dissimilar) nontargets.

The stimuli comprised a set of 48 novel computer-generated 3D objects. There were 12 target objects and 36 nontargets (distracters) varying in visual similarity to the targets (see Figure 1). Each stimulus comprised a unique spatial configuration of four different volumetric parts. The parts were defined by variation among nonaccidental properties (NAPs) comprising: edges (straight vs. curved), symmetry of the cross section, tapering (colinearity), and aspect ratio (Biederman, 1987). The object models were produced using Strata 3D CX software (Strata, U.S.A.), then rendered in Matlab using a stereo camera rig programmed with custom code. To create the stereo images left and right eye images were rendered without ‘toeing in’ using an Inter Pupillary Distance (IPD) of 62 mm. In both mono and stereo viewing conditions, participants wore polarized 3D glasses to view the stimuli presented on a passive interleaved 3D stereo monitor (60Hz 27” AOC 3D monitor (D2769VH), resolution = 1920x1080 pixels). In the stereo condition, participants viewed objects rendered from two viewpoints (left eye and right eye). In the (bi-) mono condition, participants viewed the objects with the same (right eye) rendered image presented to both eyes.[Fig-anchor fig1]

The stimuli were then normalized in size across objects to sustain in average on screen size of 17° × 17°). All stimuli were rendered using a mustard yellow color: R = 227, G = 190, B = 43, and presented on a white background to facilitate figure/ground segmentation. Object models were rendered with shading using a single top-left light source but without (internal or external) cast shadow (Leek et al., 2015).

For each of the 12 target objects, 3 corresponding nontargets were designed: one variation was composed of the same parts arranged in a different spatial configuration (SD – *S*ame Parts/*D*ifferent spatial configuration – ‘locally similar’); a second variation was composed of different parts arranged in the same configuration as the target (DS – *D*ifferent parts/*S*ame spatial configuration – ‘globally similar’); finally, in a third variation comprised different parts and spatial configuration (DD – *D*ifferent parts/*D*ifferent spatial configuration – ‘Dissimilar’). Each object was rendered at six different viewpoints varying by 60 degree rotations in depth around a vertical axis perpendicular to the line of sight.

Measures of target/nontarget image similarity using three models based on (a) Pixel overlap, (b) Gabor filter bank, and (c) HMAX - C1 output layer (Serre, Oliva, & Poggio, 2007) were computed on the 2D mono stimulus images using the Matlab Image Similarity Toolbox (Seibert & Leeds https://github.com/daseibert/image_similarity_toolbox). In the toolbox, the pixel overlap model computes the sums of squared differences in pixel intensity values between images. The Gabor filter bank model projects the image onto a Gabor wavelet pyramid as a model of V1 orientation selectivity (Kay, Naselaris, Prenger, & Gallant, 2008), using filters spanning eight orientations, four sizes (image %), and *x*, *y* positions. The Euclidian distance between the resulting vector of filter responses is compared between images. The HMAX model is based on the C1 output layer of the hierarchical feed-forward image classification model of Serre et al. (2007). We use this model to provide an estimate of image-based stimulus similarity between target and nontarget conditions. Table 1 shows the mean normalized similarity values of the three models for both target versus SD (locally similar), DS (globally similar) and DD (dissimilar) distracter image contrasts between trained and untrained viewpoints. A 2 (Viewpoint: Trained, untrained) × 3(Stimulus type: SD; DS; DD) × 3 (Model: pixel overlap; HMAX; Gabor) repeated measures ANOVA, showed no significant main effects. However, there was an interaction between Stimulus type and Model, *F*(4, 44) = 3, *p* = .029. Post hoc analyses showed that there were no differences between stimulus types for the pixel overlap or Gabor models. For HMAX there was a significant difference between SD (locally similar) and DS (globally similar) stimulus types (*p* = .02) driven by the lower mean (normalized) similarity values for trained views of target/DS (globally similar) relative to either target/SD (locally similar) or target/DD (dissimilar) stimulus contrasts.[Table-anchor tbl1]

**Figure 2 fig2:**
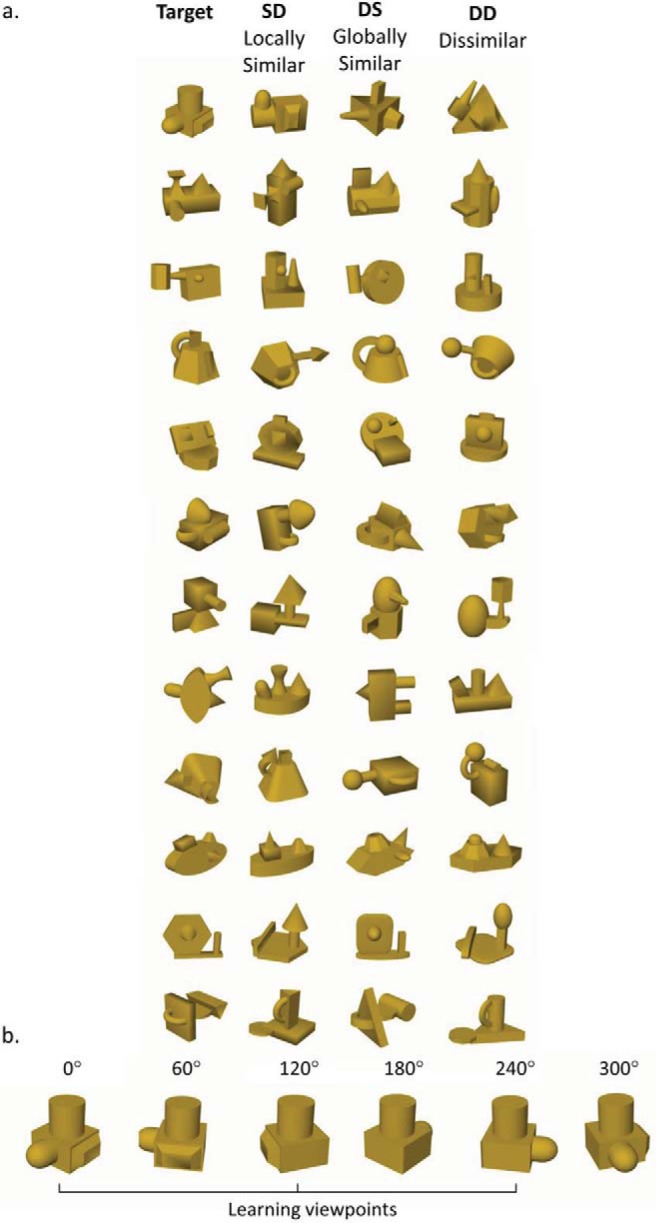
(a) All 12 target objects used in the study, with three distractor objects: SD (locally similar); DS (globally similar); DD (dissimilar). (b) One target object at the three learning (0°; 120°; 240°) and additional three test phase viewpoints (60°; 180°; 300°).

A 2 (Display: mono/stereo) × 4 (Stimulus type: Target, SD (locally similar), DS (globally similar, DD (dissimilar)) mixed factorial design was used, with Display as a between-subjects factor and Stimulus type as a within-subjects factor. Participants were randomly allocated to either the mono or stereo display group. There were 20 participants in each group. The stereo display group completed a verification task to assess their ability to fuse stereo images using interleaved polarized displays. During this task they were seated 60 cm from the screen and shown a random-dot stereogram with an embedded figure eight that was only perceivable with stereo fusion using polarized glasses. Participants were asked to report what they saw. All participants correctly reported the embedded stereo figure. The main study comprised two phases: learning and test. One group completed both the learning and test phases in mono. The other group completed both the learning and test phases in stereo. This aspect of the design ensured that any observed differences between the viewing conditions during the test phase cannot be due to mismatches in stimulus presentation formats between the learning and test phases. During the learning phase for both Groups 12 objects were memorized. In the learning phase each target was seen at three viewpoints distinguished by rotations of 120 degrees around a vertical (y) axis defined with reference to the object—see Figure 2. In the test phase, each target and nontarget was seen from six different viewpoints distinguished by 60 degree rotations around the *y* axis. In the learning phase each target was shown at each of three viewpoints three times. In the test phase, the 12 targets were presented at each of six viewpoints three times (216 target trials in total). There were also 36 nontargets (three distracters for each of the 12 targets). Each nontarget was presented once at each of the six test viewpoints (six trials per nontarget = 216 nontarget trials in total, 72 trials per nontarget condition). In total there were 432 trials in the test phase comprising equal numbers of target and nontarget trials. Trial order was randomized.[Fig-anchor fig2]

### Procedure

#### Learning phase

During the learning phase participants in both the stereo and mono groups wore polarized glasses but viewed stereo or mono images depending on the group assignment. The learning phase comprised three identical training sessions conducted over three days in separate training sessions. The purpose of the learning phase was for participants to memorize each of the 12 targets, and an associated unique stimulus number. Only participants who were able to identify targets to a criterion level of 80% after the three training sessions proceeded to the test phase. Each training session comprised a memorization stage and a verification stage. During the memorization stage target objects were presented centrally (duration = 3s) on a computer monitor sequentially at three different training viewpoints denoted 0°, 120° and 240° (see Figure 2). Target presentation was preceded by an identification number (1–12). Target identification numbers were randomly assigned across the target set but were the same for all participants. There were 36 trials (12 objects × 3 viewpoints) in each block of memorization trials. After the memorization phase, participants completed a verification task in which the 12 targets were shown randomly, one-at-a-time and for unlimited duration (until response), at each of the three viewpoints. After each stimulus, participants provided the associated target number via a key press on a standard PC keyboard. Feedback was given via a ‘Correct’ or ‘Incorrect’ message displayed centrally on the monitor. The memorization and verification tasks were repeated three times per training session (9 times across the three training sessions). All participants completed all three training sessions (regardless of whether they reached criterion accuracy earlier).

#### Test phase

**Figure 3 fig3:**
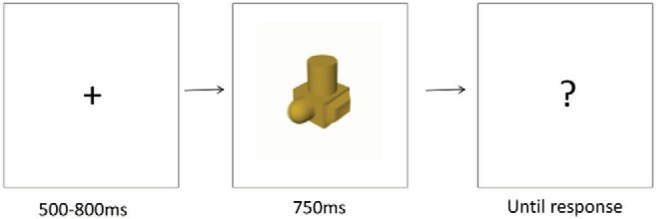
An illustration of the trial sequence comprising: (1) jittered fixation from 500-800 ms, (2) stimulus (target or nontarget) presentation for 750 ms, (3) response prompt.

During the test phase, participants in both the stereo and mono groups wore polarized glasses but viewed stereo or mono images depending on the group assignment. After the participants had completed three training sessions and had achieved the criterion level of performance in the learning phase, they completed the test phase involving a recognition memory task. The final training session of the learning phase was completed immediately before the test phase. EEGs were recorded during the test phase (see below). Each trial involved presentation of one stimulus (either a target or nontarget) at one of six viewpoints. At the start of each trial a small central fixation cross was presented in the center of the monitor at 0.7° of visual angle. The duration of the fixation cross was jittered randomly in 50 ms increments between 500 and 800 ms. Following onset of the fixation marker the test stimulus was shown for 750 ms. This stimulus was replaced by a response screen (centrally presented question mark). All trial events were separated by an interstimulus interval of one screen refresh (17 ms). Participants were instructed to respond via a button press using a standard PC keyboard (“1” for target and “2” for nontarget—with the fore and middle fingers of the right hand respectively for all participants) indicating whether the stimulus shown was one of the 12 objects that they had previously memorized regardless of its orientation. They were alerted to the fact that the stimuli could be presented at previously seen and novel viewpoints. Participants could only respond following onset of the response screen, and not during presentation of the stimulus. This was done to help reduce potential motor response artifacts in the EEG. The response screen remained until a response was given (see Figure 3). The intertrial interval was a blank screen presented for 1000 ms. For the behavioral data the dependent measure was response accuracy. RTs were not collected because keyboard responses were only acquired from the onset of the response screen. This was done to reduce motor artifacts in the ERPs associated with the stimulus event.[Fig-anchor fig3]

#### Electrophysiological recording and processing

The electroencephalograph (EEG) was recorded continuously through 128 electrodes placed on an ECI cap (Electro-Cap International, Ohio, U.S.A.) using the Active-Two Biosemi EEG system (Biosemi V.O.F Amsterdam, Netherlands). Eye movements and blinks were corrected using the ICA protocol in Analyser 2 software and segmented data was then visually inspected with trials containing artifacts rejected. Epochs that contained muscle or skin potential artifacts were rejected. Only trials on which participants gave a correct response were included. The mean number of correct trials per subject after artifact rejection was: 189.25 (SS/target), 62.61 (SD/locally similar), and 67.61 (DS/globally similar) and 67.82 (DD/dissimilar). Activity from all electrodes was sampled at a rate of 1024Hz. Offline 30 Hz low pass and 0.1 Hz high-pass filters were applied to the data. Data were rereferenced to an average reference which was then used to generate the grand averages. We used a 100-ms prestimulus interval for the baseline correction. Continuous recording took place during the test phase and trials were epoched/segmented from −100 ms to stimulus offset (750 ms). All ERP data acquired from onset of the response prompt were discarded.

#### EEG analyses

Four early visual evoked potential components P1, N1, P2, and an N2–P3 complex were identified based on the topography, global field power (GFP) deflection and latency characteristics of the respective grand average ERPs time-locked to stimulus presentation. Preliminary epochs of interest for each component were defined based on deflection extrema in the mean local field power (e.g., Brunet, Murray, & Michel, 2011; Lehmann & Skrandies, 1980; Murray, Brunet, & Michel, 2008). Peak detection was time-locked to the electrode of maximal amplitude for each component. The latency of peak amplitude was used to define epochs for analyses of four components: Mono - P1 (85–125 ms; Peak latency (A10) = 105 ms; N1 (145–185 ms; Peak latency (B7) = 165 ms); P2 (200–240 ms; Peak latency (A8) = 220 ms); N2–P3 complex (285–385 ms; Peak latency (A8) = 335 ms); Stereo - P1 (90–130 ms; Peak latency (B7) = 110 ms); N1 (150–190 ms; Peak latency (A11) = 170 ms); P2 (195–235 ms; Peak latency (A8) = 215 ms); N2–P3 (260–360 ms; Peak latency (A7) = 310 ms).

Symmetrical clusters were extracted over the left (LH) and right (RH) hemispheres comprising nine spatially adjacent posterior electrodes: RH: A32, B3, B4, B5, B6, B7, B8, B10, B11, and LH: A5, A6, A7, A8, A9, A10, A11, D31, and D32, which correspond with electrode locations CP2, P4, P6, P8, PO8 and CP1, P3, P5, P7, PO7 of the extended 10–20 system. These electrode clusters formed the regions-of-interest (ROIs) for the subsequent analyses of contrasts between stimulus conditions. Standard waveform analyses were based on the amplitude data as a measure of differential ERP sensitivity to 3D shape similarity between mono and stereo viewing. Mean amplitudes were analyzed using the General Linear Model by way of ANOVA. Greenhouse-Geisser corrections were applied to all analyses of ERP data. Corrected degrees of freedom are reported where applicable. An a priori alpha level of .05 (two-tailed) was adopted. Exact *p* values are reported (*p* = x) except where *p* < .001.

#### Mass univariate analyses

Mass Univariate analyses (Groppe, Urbach, & Kutas, 2011; Guthrie & Buchwald, 1991; Murray et al., 2008) were used to complement the standard waveform analyses. This involved using pair wise, frame-by-frame, repeated measures *t* tests across all 128 electrodes. An a priori criterion for significance was adopted in which a threshold of *p* < .01 (two-tailed) must be attained for at least 12 consecutive time frames in at least 5 neighboring electrodes over time windows of 150 ms (Guthrie & Buchwald, 1991). For this purpose, the mass univariate analyses were conducted on 150-ms bins (0–150 ms; 151–300 ms; 301–450 ms) encompassing the P1, N1, P2, and N2/P3 components.

## Results

### Behavioral Results

Accuracy data were log transformed prior to statistical analyses.

### Learning Phase

A 3(Training day) × 2(Display: mono; stereo) mixed ANOVA, with Display as a between subjects factor showed significant main effects of Training day, *F*(2, 60) = 58.06, *p* < .001, with accuracy (% correct) increasing over time, from day one (*M* = 69.48, *SD* = 17.38) to two (*M* = 94.71, *SD* = 8.06), *p* < .001, and two to three (*M* = 98.09, *SD* = 4), *p* = .006. There were no differences between mono and stereo display groups and all participants passed criterion by the end of the third training session.^2,^^3^

### Test Phase

**Figure 4 fig4:**
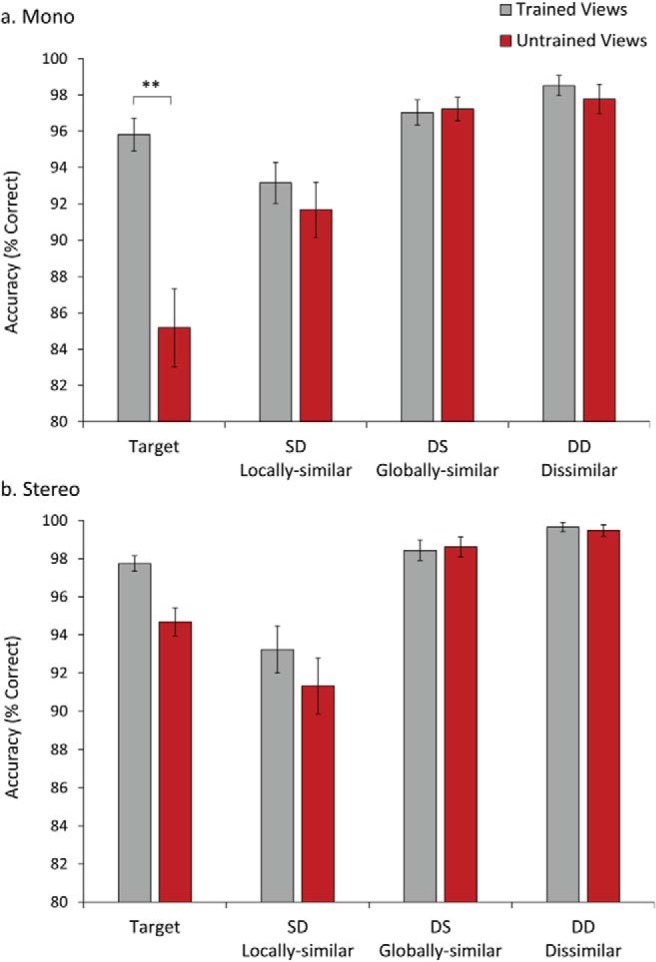
Accuracy for targets in mono and stereo viewing conditions in the test phase. Bars show standard error.

Figure 4 shows mean percent correct responses per condition. The data were analyzed using a 4 (Stimulus type: Target; SD [locally similar]; DS [globally similar]; DD [dissimilar]) × 2 (Stimulus viewpoint: trained/untrained) × 2 (Display: mono/stereo) mixed ANOVA, with Display as a between subjects factor. There were significant main effects of Stimulus type, *F*(3, 90) = 13.5, *p* < .001, and Stimulus viewpoint, *F*(1, 30) = 10.41, *p* = .003, with higher overall accuracy for trained (*M* = 97.05%, *SD* = 2.65) than untrained (*M* = 95.4%, *SD* = 3.42) viewpoints. There was also a significant three-way interaction, *F*(3, 87) = 3.19, *p* = .027. To investigate this further we analyzed mono and stereo data separately using 4 (Stimulus type) × 2 (Stimulus viewpoint) repeated measures ANOVAs. For the mono viewing group, there was an interaction between Stimulus type and Stimulus Viewpoint, *F*(3, 45) = 5.9, *p* = .002. This derived from significantly higher accuracy for trained than untrained viewpoints for target stimuli, *p* = .003 (see Figure 4). In contrast, for the stereo viewing group there were no significant main effects or interactions. Finally, accuracy for targets presented at untrained views was higher for stereo (*M* = 94.68%, *SD* = 5.09) than mono (*M* = 85.19%, *SD* = 14.46) displays (*p* = .035). This pattern of results is consistent with a stereo advantage in view generalization for targets between trained and untrained views.[Fig-anchor fig4]

### Analyses of ERP Data

The aims of these analyses were as follows: (a) to determine whether the ERP showed sensitivity to the manipulation of stereo and mono viewing; (b) to establish whether the ERPs were differentially sensitive to target/nontarget shape similarity defined by either shared local parts or global 3D spatial configuration; and (c) to determine whether differential perceptual sensitivity to these shape attributes was modulated by mono versus stereo viewing.

#### ERP analyses I: Perceptual sensitivity to stereo/mono presentation

**Figure 5 fig5:**
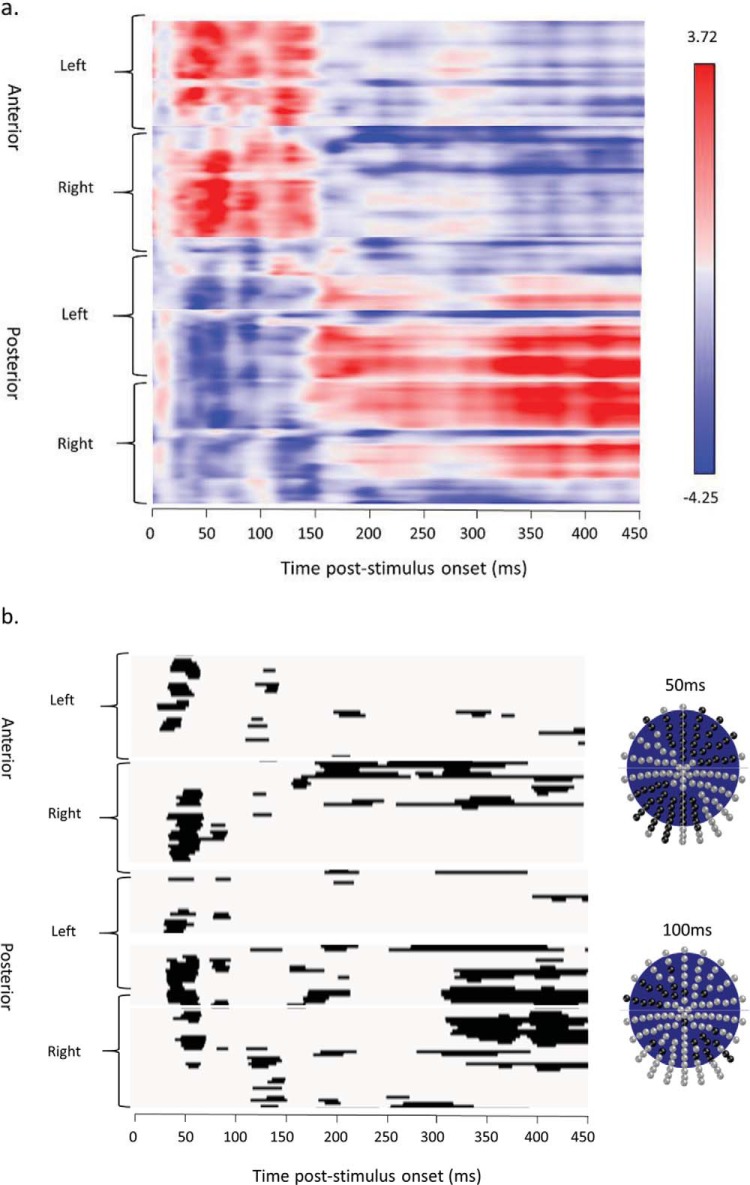
Raster plots of mass univariate contrasts for mono versus stereo presentation for anterior and posterior left and right hemisphere electrodes (*y* axis), across time frames from 0–450 ms poststimulus onset (*x* axis); (a) shows a color-coded t-map displaying the polarity of contrasts and max/min t values; (b) thresholded plot showing significant pairwise contrasts (*p* < .01). The electrode montages show the electrodes significant at *p* < .01 at 50 ms (above) and 100 ms (below) poststimulus onset in black.

We first wanted to determine whether our display manipulation of stereo versus mono presentation was sufficient to induce a measurable early perceptual sensitivity in visual evoked potentials. Mass univariate analyses were used to identify a temporal marker defining the earliest time point of differential ERP sensitivity to mono versus stereo viewing. A point-wise mass univariate contrast between the mono and stereo viewing across all conditions revealed differences in the ERP from around 50 ms poststimulus onset over a large group of posterior, temporal-occipital and anterior leads. This difference was sustained during the P1 component over left occipital and some frontal electrodes (see Figure 5). These analyses confirm an early perceptual sensitivity to mono versus stereo viewing.[Fig-anchor fig5]

#### ERP analyses 2: Perceptual sensitivity to 3D shape similarity as a function of mono/stereo viewing

Our next goal was to establish whether perceptual processing of object shape resulted in differential sensitivity to local parts and global 3D shape configuration as a function of mono versus stereo viewing. To do so we conducted both standard waveform analyses and mass univariate contrasts.

### Standard Waveform Analyses

#### P1

**Figure 6 fig6:**
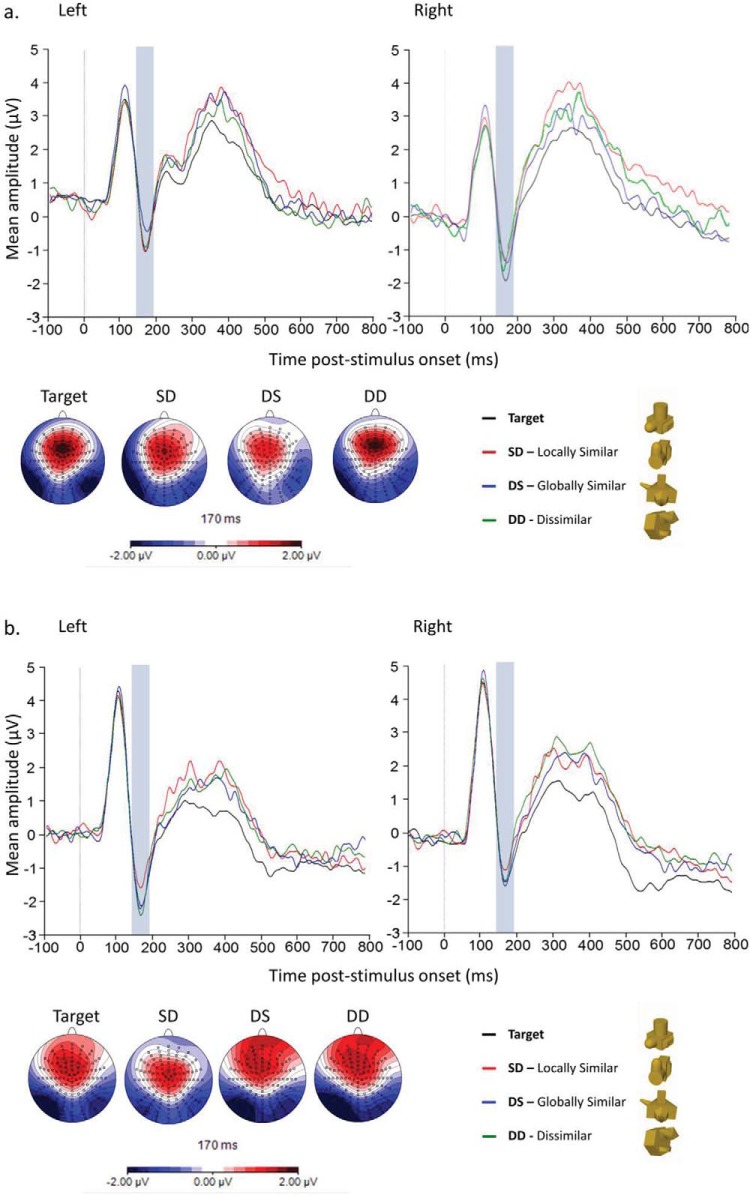
Grand average waveforms for the N1 component (blue highlight) across conditions at the electrode cluster encompassing P7 and PO7 (left hemisphere) and P8 and PO8 (right hemisphere) for (a) Mono and (b) Stereo viewing groups.

This was defined by a 40-ms time window (85–125 ms for mono and 90–130 ms for stereo). A 4 (Stimulus type: Target; SD [locally similar]; DS [globally similar]; DD [dissimilar]) × 2 (Laterality) × 2 (Display: mono/stereo) mixed ANOVA, with Display as a between subjects factor, showed a main effect of Display, *F*(1, 30) = 5.41, *p* = .028, with higher amplitudes (μV) for stereo (*M* = 4.03, *SD* = 0.54) than mono viewing (*M* = 2.72, *SD* = 0.15; see Figure 6a and 6b). There was also a main effect of Laterality, *F*(1, 30) = 8.28, *p* = .007, with greater amplitudes on the right (*M* = 3.63, *SD* = 0.99) than left (*M* = 3.12, *SD* = 0.46) hemisphere electrodes. No other main effects or interactions were significant.[Fig-anchor fig6]

#### N1

This was defined by a time window of 145–185 ms for mono, and 150–190 ms for stereo viewing. A 4 (Stimulus type: Target; SD [locally similar]; DS [globally similar]; DD [dissimilar]) × 2 (Laterality) × 2 (Display: mono; stereo) mixed ANOVA, with Display as a between subjects factor, showed a significant three-way interaction, *F*(2.82, 84.51) = 2.98, *p* = .044. No other main effects or interactions were significant. As can be seen in Figure 6 this interaction derives from the contrasting patterns of amplitude modulation in the SD (locally similar) and DS (globally similar) conditions between mono and stereo viewing. To investigate this further we conducted two separate 4 (Stimulus type: Target; SD [locally similar]; DS [globally similar]; DD [dissimilar]) × 2 (Laterality) repeated measures ANOVAs for the mono and stereo display conditions.

For the mono condition (see Figure 6a), there was a main effect of Stimulus type, *F*(2.27, 34.05) = 3.85, *p* = .03, driven by a significant difference between the target and DS (globally similar) nontargets, *p* = .02, with greater negativity for targets (*M* = −0.75, *SD* = 0.26) than DS (globally similar) (*M* = −0.23, *SD* = 0.25) stimuli. No other main effects or interactions were significant. In contrast, for the stereo condition (see Figure 6b) there was a significant interaction between Stimulus type and Laterality, *F*(2.76, 41.47) = 2.88, *p* = .046. Post hoc contrasts showed a significant difference between the targets and SD (locally similar) nontargets in the left hemisphere only, *t*(15) = 2.29, *p* = .036, with increased negativity for targets (*M* = −1.39 *SD* = 0.46) compared with SD (locally similar) (*M* = −0.97, *SD* = 0.43) stimuli. No other main effects or interactions were significant.

#### P2

This was defined by a time window of 200–240 for mono and 195–235 ms for stereo viewing. A 4 (Stimulus type: Target; SD [locally similar]; DS [globally similar]; DD [dissimilar]) × 2 (Laterality) × 2 (Display: mono; stereo) mixed ANOVA, with Display as a between subjects factor showed that no main effects or interactions were significant.

#### N2–P3 complex

**Figure 7 fig7:**
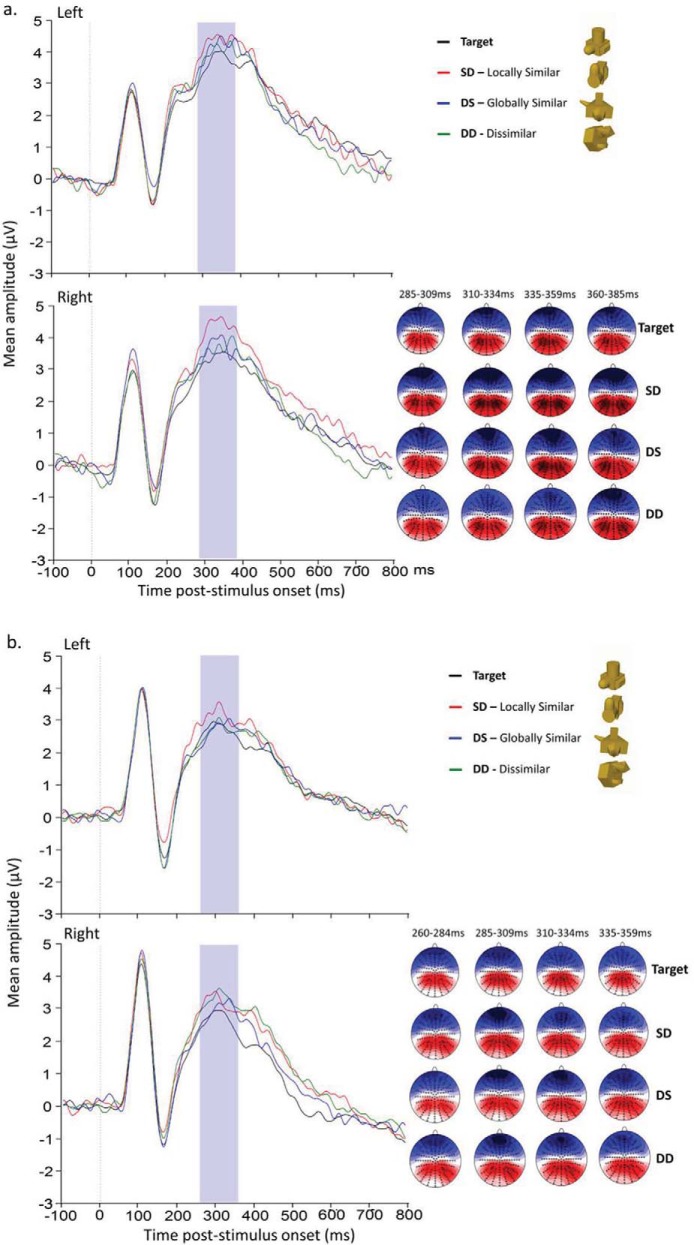
N2–P3 grand average waveforms (highlighted in blue shaded area) for (a) Mono and (b) Stereo viewing groups for all conditions at the electrode clusters encompassing P3 and CP1 (left hemisphere) and P4 and CP2 (right hemisphere).

The N2–P3 complex was defined by a time window of 285–385 ms for mono and 260–360 ms for stereo viewing. A 4 (Stimulus type: Target; SD [locally similar]; DS [globally similar]; DD [dissimilar]) × 2 (Laterality) × 2 (Display: mono; stereo) mixed ANOVA, with Display as a between subjects factor showed a significant main effect of Stimulus type, *F*(2.48, 74.24) = 2.97, *p* = .046. There was also a significant three-way interaction, *F*(2.82, 84.51) = 3.48, *p* = .022. There were no other significant main effects or interactions. To investigate this further we analyzed mono and stereo data separately using 4 (Stimulus type) × 2 (Laterality) repeated measures ANOVAs. For the mono viewing group (Figure 7a) there were no significant main effects or interactions. In contrast, for the stereo viewing group (Figure 7b) there was a significant interaction between Stimulus type and Laterality, *F*(2.76, 41.47) = 4.51, *p* = .009. Planned comparisons showed that there were no differences between stimulus types in the left hemisphere, but in the right hemisphere mean amplitude for targets was lower than SD (*p* = .022), DS (*p* = .024) and DD (*p* = .002). No other main effects or interactions were significant.[Fig-anchor fig7]

### Further Analyses I: Mass Univariate Contrasts Across All 128 Electrodes

**Figure 8 fig8:**
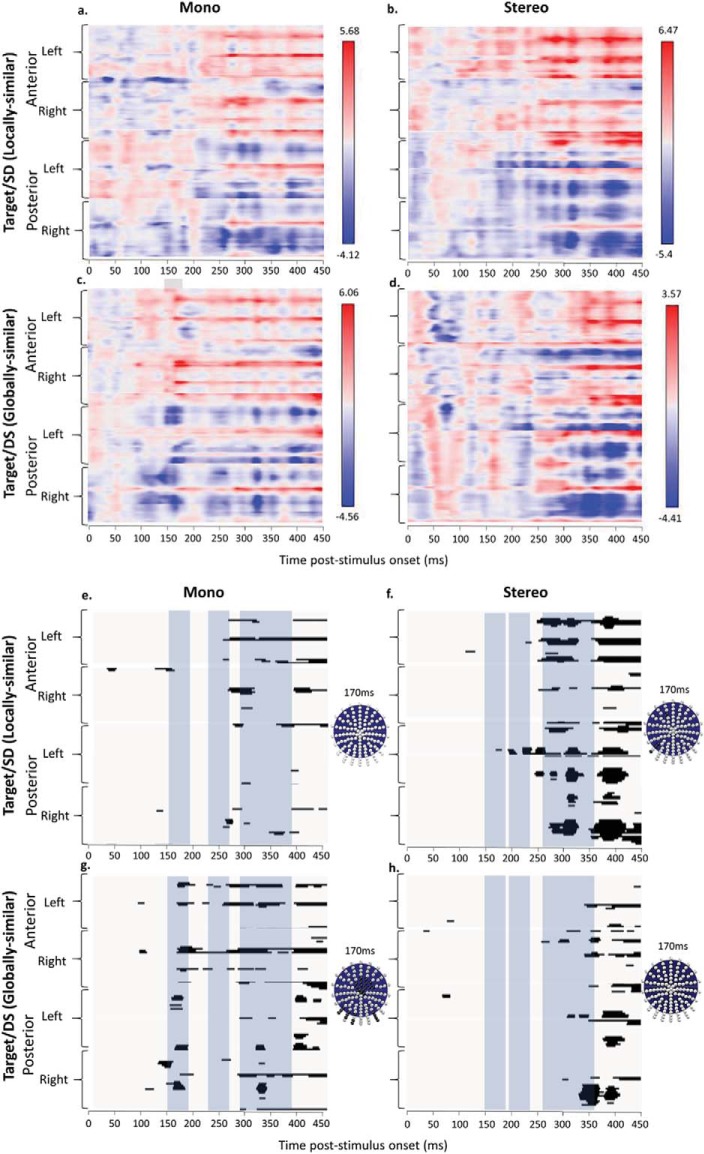
Raster plots of mass univariate contrasts for (a/e) Mono Target-SD (Locally similar); (b/f) Stereo Target-SD (Locally similar); (c/g) Mono Target-DS (globally similar) and (d/h) Stereo Target-DS (globally similar). Posterior/anterior and right/left electrodes are shown (*y*-axis) across time frames from 0-450 ms poststimulus onset; (a–d) show color-coded t-maps displaying the polarity of contrasts and max/min t values; (e–h) thresholded plots showing significant pairwise contrasts (*p* < .01). The electrode montages show the electrodes significant at *p* < .01 at 50 ms (above) and 100 ms (below) poststimulus onset in black for each contrast. The blue highlighted areas show the N1, P2, and N2/P3 components.

Mass univariate analyses were used to complement our standard waveform analyses of the effects of mono and stereo viewing on the discrimination between targets and critical SD (locally similar) and DS (globally similar) nontargets. Unlike the standard analysis, the mass univariate approach allows us to examine the patterns of contrasts between conditions across all 128 electrodes (rather than restricting the analysis to the 9 electrode cluster in each hemisphere). The temporal distributions of these contrasts across all 128 electrodes for mono viewing are shown in Figure 8a–8h.[Fig-anchor fig8]

**Figure 9 fig9:**
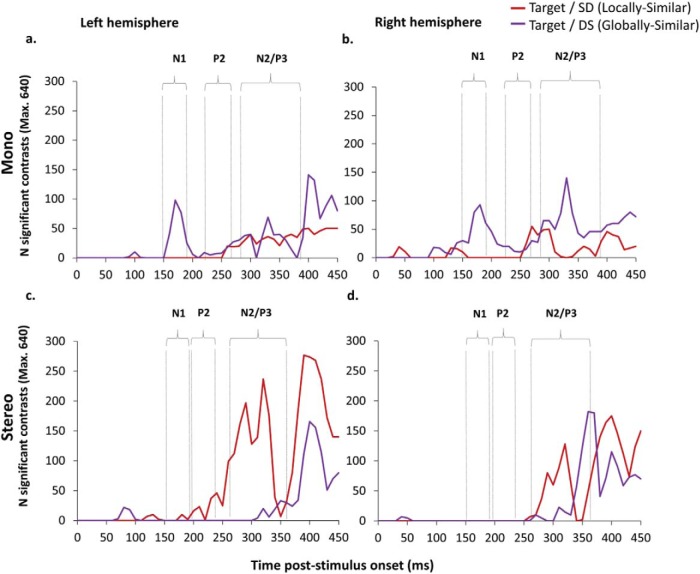
Time series distribution showing the frequency of significant difference contrasts from the mass univariate analysis between 0 and 450 ms. Contrasts shown are between Target and SD (locally similar; red) and Target and DS (globally similar; purple) for both mono in the (a) left and (b) right hemispheres and stereo in the (c) left and (d) right hemispheres.

These mass univariate contrasts show the differential sensitivity between targets and SD/DS nontargets for mono and stereo viewing in the N1, P2 and N2/P3 components. A time series plot of the frequency distribution of significant differences is shown in Figure 9. These data were analyzed as a nonparametric time-series using the Friedman test. For the N1 during mono viewing there was a higher frequency of significant differences between targets and DS (globally similar) nontargets in both the left, χ^2^(1) = 4, *p* = .046 and right hemispheres, χ^2^(1) = 5, *p* = .025. For stereo viewing there was a higher frequency of significant differences between targets and SD (locally similar) nontargets in the left hemisphere only, χ^2^(1) = 4, *p* = .046. The same pattern for stereo viewing was also found during the P2, χ^2^(1) = 4, *p* = .046, but there was no significant differences for the mono group. The N2/P3 component also showed a striking contrast in perceptual sensitivity to SD (locally similar) and DS (globally similar) nontargets between mono and stereo viewing. For mono viewing there was a higher frequency of significant differences between targets and DS (globally similar) nontargets in the right hemisphere, χ^2^(1) = 10, *p* = .002. The opposite pattern was found for stereo viewing with a higher frequency of significant differences between targets and SD (locally similar) nontargets in the left hemisphere, χ^2^(1) = 6.4, *p* = .011.[Fig-anchor fig9]

### Further Analyses II: Effects of Training Viewpoint

**Figure 10 fig10:**
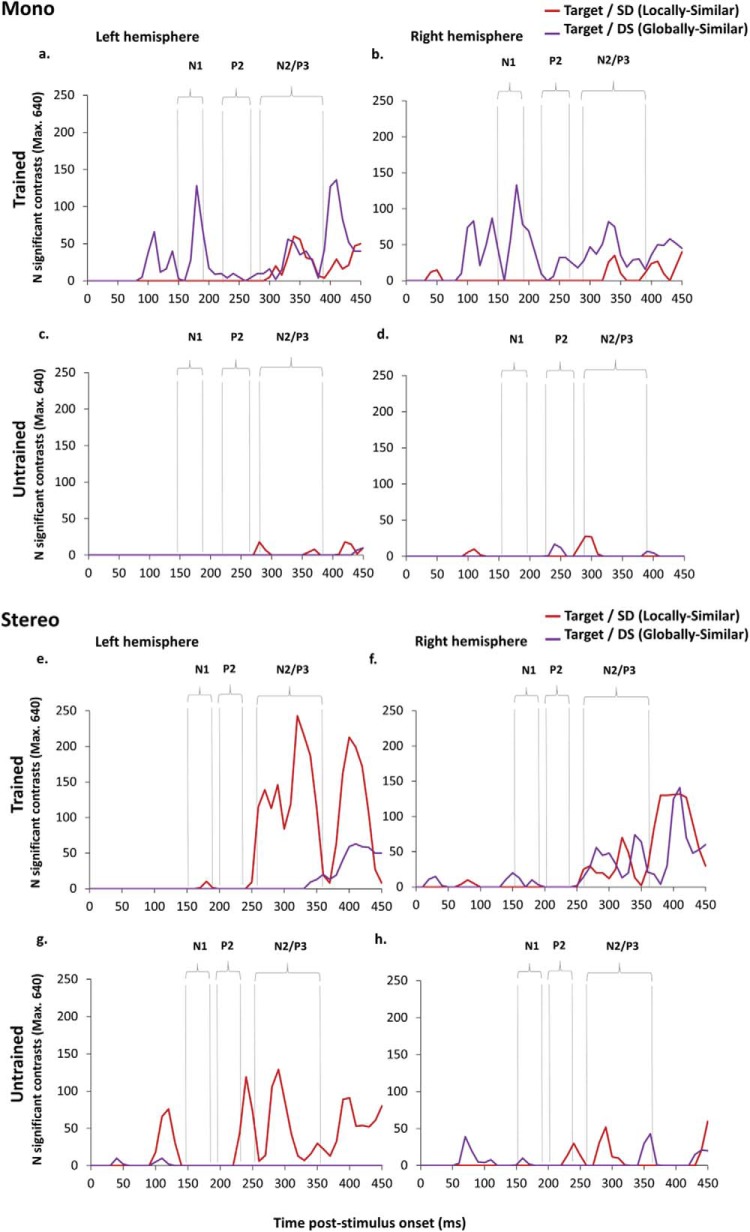
Time series distribution showing the frequency of significant difference contrasts from the mass univariate analysis between 0 and 450 ms. Contrasts shown are between target and SD (locally similar) in red and target and DS (globally similar) nontargets in purple for mono (a-d)/stereo (e-h) viewing, left and right hemispheres and trained versus untrained views.

The analyses so far show differential sensitivity to SD (locally similar) and DS (globally similar) nontargets between mono and stereo viewing. In brief, during mono viewing there is a greater response modulation to target versus DS (globally similar) nontargets in both the left and right hemisphere that begins during the N1 and continues into the later N2/P3 component. During stereo viewing, there is a greater response modulation to target versus SD (locally similar) nontargets that is predominant in the left hemisphere and which begins during the N1 but only peaks during the later N2/P3. In a final analysis, we wanted to examine whether these differential response patterns are modulated by viewpoint familiarity; that is, whether they generalize across image classification at trained and untrained views. Figure 10 shows a time series plot of the frequency distribution of significant differences between target and nontarget conditions for trained and untrained viewpoints. The data were analyzed as a nonparametric time-series using the Friedman test. For the mono viewing group the higher frequency of significant differences between target and DS (globally similar) distracters in the left and right hemispheres during the N1 was found for trained viewpoints but did not generalize to untrained viewpoints (LH: χ^2^(1) = 4, *p* = .046, RH: χ^2^(1) = 4, *p* = .046). In contrast, for the stereo viewing group, there were no differences between trained and untrained viewpoints at the N1. For the mono group at the N2/P3, however, there was a higher frequency of significant differences between target and SD (locally similar) distracters for trained than untrained viewpoints in the left hemisphere, χ^2^(1) = 6.4, *p* = .011. There was also a higher frequency of differences between target and DS distracters in the left and right hemispheres for trained than untrained viewpoints (LH: χ^2^(1) = 10, *p* = .002; RH: χ^2^(1) = 10, *p* = .002). For the stereo group, there was a higher frequency of significant differences between target and SD (locally similar) distracters for trained than untrained viewpoints in the left hemisphere, χ^2^(1) = 6.4, *p* = .011 and a higher frequency of differences between target and DS (globally similar) distracters for trained than untrained viewpoints in the right hemisphere, χ^2^(1) = 6.4, *p* = .011.[Fig-anchor fig10]

## Discussion

The main findings can be summarized as follows: First, the behavioral data provided evidence for an advantage in view generalization for stereo over mono displays. This was shown by higher accuracy in target classification of untrained views for stereo displays. Second, the ERP data showed differential amplitude responses to mono versus stereo viewing as early as 50–100 ms poststimulus onset, with higher amplitudes on the P1 component for stereo displays. Third, we observed differential amplitude modulations of evoked potentials to targets and nontargets defined by shared parts (SD; locally similar) or shared spatial configuration (DS; globally similar) starting at the N1 component between 145 and 200 ms poststimulus onset. N1 amplitudes for mono displays showed greater differential sensitivity to DS (globally similar) nontargets. For stereo displays, there was a greater differential amplitude modulation for SD (locally similar) nontargets in left hemisphere electrodes. Fourth, a pattern of differential amplitude modulation was also found at the later N2/P3 component around 260–385 ms poststimulus onset. This was most clearly shown in the mass univariate analysis. For mono viewing, there was a higher frequency of significant differences between targets and DS (globally similar) nontargets. For stereo viewing, there was a higher frequency of significant differences between targets and SD (locally similar) nontargets. Fifth, under mono viewing, the differential sensitivity to DS (globally similar) nontargets was found for trained but not untrained views. In contrast, the amplitude sensitivity in stereo viewing to SD (locally similar) nontargets was found with both trained and untrained views.

These new empirical findings have several important implications for models of object recognition. First, the results provide new evidence that the representation of complex 3D object shape involves the specification of higher-order part structure and 3D part configuration. This is shown by the differential sensitivity in the ERPs to shape differences between targets and nontargets defined by either shared local parts or 3D shape configuration. These differences emerged during the N1 component between approximately 145–200 ms poststimulus onset, and were also found during the N2/P3 component around 260–385 ms poststimulus onset. This finding is consistent with theoretical models, and other supporting empirical evidence, that the perceptual representation of complex 3D object shape involves the specification of higher-order part structure and global 3D spatial configuration (e.g., Arguin & Saumier, 2004; Behrmann et al., 2006; Behrmann & Kimchi, 2003; Biederman, 1987; Hummel & Stankiewicz, 1996; Marr & Nishihara, 1978). The results challenge theoretical models which do not attribute functional significance to these properties of object shape representations - including the hierarchical, feed-forward HMAX deep (i.e., multilayer) network architecture (e.g., Riesenhuber & Poggio, 1999; Serre et al., 2007), and others (e.g., Bulthoff & Edelman, 1992; Chan et al., 2006; Khaligh-Razavi & Kriegeskorte, 2014; Krizhevsky et al., 2012; Li & Pizlo, 2011; Li et al., 2009; Pizlo, 2008).

Second, the results also provide new evidence that the recognition of complex 3D object shape can be modulated by stereo visual input. This was shown in both the behavioral and ERP data patterns. Behaviorally, we found an advantage for object recognition under conditions of stereo viewing in relation to classification accuracy for targets presented at previously untrained views. This observation adds to a growing body of behavioral evidence that stereo input can facilitate 3D object recognition—at least under some conditions (e.g., Bennett & Vuong, 2006; Burke, 2005; Burke et al., 2007; Chan et al., 2006; Edelman & Bülthoff, 1990; Hong Liu et al., 2006; Lee & Saunders, 2011; Rock & DiVita, 1987; Simons et al., 2002). According to Cristino et al. (2015), stereo input provides additional cues to 3D object shape including, for example, the specification of surface slant, curvature polarity and 3D part configuration. We also found differential modulation of ERP amplitudes during mono and stereo viewing as a function of target/nontarget shape similarity. Notably, we found evidence for differential modulation of ERP amplitudes under mono and stereo viewing for DS (globally similar) and SD (locally similar) distractors. This shows that stereo viewing can modulate perceptual processing of different attributes of 3D shape—contrary to the predictions of theoretical models that do not attribute functional significance to stereo information in the derivation of 3D object representations (e.g., Bulthoff & Edelman, 1992; Chan et al., 2006; Li & Pizlo, 2011; Li et al., 2009; Pizlo, 2008; Riesenhuber & Poggio, 1999; Serre et al., 2007). One interpretation of the results is that stereo viewing enhances processing of information about the 3D spatial configuration of object parts, and that this information facilitates the classification of SD (locally similar) distracters as nontargets on the basis of their distinct global 3D spatial configuration. In contrast, under conditions of mono viewing, we found early differential sensitivity to DS (globally similar) distracters that shared spatial configuration but not local parts (that is, where targets and distractors can be differentiated on the basis of distinct local parts). This raises the possibility that, in the absence of stereo input (as is the case in most previous empirical studies of object processing), the perceptual analysis of 3D object shape is weighted toward differences in 2D local shape attributes. Furthermore, the enhanced processing of local part structure did not generalize to untrained views, suggesting that under monocular viewing conditions object shape processing may be weighted toward an ‘image-based’ processing strategy. Taken together, these findings suggest that mental representations of 3D object shape in human vision are rich in structure, encoding both 2D image-based local features, and 3D shape properties, broadly consistent with a ‘hybrid’ approach to object recognition mediated by representations combining both 2D and 3D object structure (Foster & Gilson, 2002; Hummel, 2013; Hummel & Stankiewicz, 1996).^4^

A recent study by Leek et al. (2016), using a sequential novel object matching task under conditions of mono viewing only, also reported early differential perceptual sensitivity to shape differences defined by either shared parts or global spatial configuration. In that work, differential sensitivity in perceptual matching of novel 3D objects was—as in the current study, found to emerge earliest on amplitude modulations during the N1 component over posterior electrodes between objects sharing either local parts or global spatial configuration. The current data extend these findings in several important ways. First, we have shown that this differential perceptual sensitivity extends to an object recognition task where observers are required to match a perceptual description of 3D object shape to a (previously learned) long-term memory representation. Second, the results also show that this differential perceptual sensitivity is modulated by mono versus stereo input—in which mono viewing enhances local differences in part structure, while stereo viewing enhances differences in global 3D spatial configuration. Third, we also found that this stereo viewing effect generalizes across changes in 3D object viewpoint, whereas perceptual sensitivity to local differences in part structure found under conditions of mono viewing were restricted to trained viewpoints.

An additional important issue arises from our observation of early perceptual sensitivity of ERPs to shape similarity between targets and distracters on the N1 component. This implies that some properties of the shape of 3D objects can modulate perceptual processing prior to recognition (e.g., Bar, 2003; Bar et al., 2006; Leek et al., 2016; Leek, d’Avosssa, Yuen, Hu et al., 2012; Lloyd-Jones, Roberts, Leek, Fouquet et al., 2012). One interpretation of this effect is that the early perceptual modulation reflects partial activation of stored (i.e., target) shape representations on the basis (in this case) of parts-based object descriptions. More broadly, this hypothesis is consistent with a conception of object shape processing that is based on parallel analyses of shape across multiple spatial scales (e.g., Bar, 2003; Bar et al., 2006; Hegdé, 2008; Heinze, Johannes, Munte, & Mangun, 1994; Heinze, Hinrichs, Scholz, Burchert, & Mangun, 1998; Navon, 1977; Peyrin, Chauvin, Chokron, & Marendaz, 2003; Peyrin, Baciu, Segebarth, & Marendaz, 2004; Peyrin et al., 2010).

Finally, one other issue merits brief discussion. Although our primary goal was to examine whether mono versus stereo visual input differentially modulates the perceptual processing of 3D object shape during recognition, we also observed an early perceptual sensitivity, and lateral asymmetry, to stereo disparity. We found the earliest differential responses to mono versus stereo input from around 50 ms poststimulus onset over a large group of posterior, temporal-occipital and anterior leads. This difference was sustained during the P1 component over left occipital and some frontal electrodes. Additionally, we also found greater P1 amplitudes for right over left hemisphere electrode sites. We have taken this to reflect early perceptual sensitivity to mono- versus stereo input in our design. One might argue that these differences do not reflect the resolution of stereo disparity per se, but rather sensitivity to the presentation of different images to the left and right eye in the stereo condition. However, if this were the case, we would expect to find differences between mono- and stereo presentation in all conditions regardless of target-distracter similarity. The observed interactions between stimulus type and viewing condition show that this was not the case.

In summary, we investigated whether stereo viewing modulates perceptual processing of 3D object shape. A recognition memory task was used in which observers were trained to recognize a subset of 3D novel objects under conditions of either mono or stereo viewing. In a subsequent test phase, they discriminated trained objects from nontargets that shared either local parts, 3D spatial configuration or neither dimension, across both previously trained and novel viewpoints. The behavioral data showed a stereo advantage for generalization between trained and untrained views. ERPs amplitudes also showed early differential sensitivity to local part, and 3D spatial configuration, similarity between targets and distracters. This occurred during an N1 component from 145–200 ms poststimulus onset and during an N2/P3 component from 260–385 ms poststimulus onset. For mono viewing, amplitude modulation during the N1 was greatest between targets and distracters with different local parts for trained views only. For stereo viewing, amplitude modulation during the N2/P3 was greatest between targets and distracters with different global 3D spatial configurations and generalized across trained and untrained views. The results show that image classification is modulated by stereo information about the local part, and global 3D spatial configuration of object shape. The findings challenge current theoretical models that do not attribute functional significance to stereo input during the computation of 3D object shape.

## Figures and Tables

**Table 1 tbl1:** Mean (SD) Normalized (0–1) Image Similarity Between Targets and Distractors (Nontargets) for the Pixel Overlap, HMAX, and Gabor Models

Model	View			
	Trained		Untrained	
	*M*	*SD*	*M*	*SD*
Pixel Overlap				
SD (Locally-similar)	.42	.19	.39	.17
DS (Globally-similar)	.34	.16	.35	.18
DD (Dissimilar)	.39	.18	.39	.14
HMAX				
SD (Locally-similar)	.31	.10	.31	.09
DS (Globally-similar)	.17	.11	.31	.09
DD (Dissimilar)	.30	.14	.28	.09
GABOR				
SD (Locally-similar)	.30	.15	.31	.13
DS (Globally-similar)	.33	.11	.35	.14
DD (Dissimilar)	.26	.12	.25	.07
*Note*. Smaller values indicate lower similarity.				

## Footnotes

1 Throughout the paper we use the term ‘mono’ to describe nonstereo ‘bimonocular’ visual input (that is, where there is no disparity between visual inputs to the left and right eye). Stereo refers to visual input with binocular disparity (i.e., different left and right eye images for a given viewpoint).

2 Nine of twenty-eight participants reached criterion accuracy after the first training session.

3 Patterns of behavioral and ERP data for the three left-handed participants and the group were the same across conditions.

4 In relation to the HMAX hypothesis in particular, it is of interest to note that in terms of image similarity, we also found lower mean (normalized) HMAX target-distractor similarity values for trained views. This could potentially have also contributed to the differential sensitivity of ERP amplitudes to DS and SD nontargets found for mono viewing consistent with an image-based processing strategy. However, this would not account for the why the opposite pattern of amplitude modulation was found with stereo input.

## References

[c1] Arguin M., & Leek E. C. (2003). Orientation invariance in visual object priming depends on prime-target asynchrony. Perception & Psychophysics, 65, 469–477. 10.3758/BF03194576 12785075

[c2] Arguin M., & Saumier D. (2004). Independent processing of parts and of their spatial organization in complex visual objects. Psychological Science, 15, 629–633. 10.1111/j.0956-7976.2004.00731.x 15327635

[c3] Ban H., & Welchman A. E. (2015). fMRI analysis-by-synthesis reveals a dorsal hierarchy that extracts surface slant. The Journal of Neuroscience, 35, 9823–9835. 10.1523/JNEUROSCI.1255-15.2015 26156985 PMC4495240

[c4] Bar M. (2003). A cortical mechanism for triggering top-down facilitation in visual object recognition. Journal of Cognitive Neuroscience, 15, 600–609. 10.1162/089892903321662976 12803970

[c5] Bar M., Kassam K. S., Ghuman A. S., Boshyan J., Schmid A. M., Dale A. M., . . . Halgren E. (2006). Top-down facilitation of visual recognition. Proceedings of the National Academy of Sciences of the United States of America, 103, 449–454. 10.1073/pnas.0507062103 16407167 PMC1326160

[c6] Behrmann M., & Kimchi R. (2003). What does visual agnosia tell us about perceptual organization and its relationship to object perception? Journal of Experimental Psychology: Human Perception and Performance, 29, 19–42. 10.1037/0096-1523.29.1.19 12669745

[c7] Behrmann M., Peterson M. A., Moscovitch M., & Suzuki S. (2006). Independent representation of parts and the relations between them: Evidence from integrative agnosia. Journal of Experimental Psychology: Human Perception and Performance, 32, 1169–1184. 10.1037/0096-1523.32.5.1169 17002529

[c8] Bennett D. J., & Vuong Q. C. (2006). A stereo advantage in generalizing over changes in viewpoint on object recognition tasks. Perception & Psychophysics, 68, 1082–1093. 10.3758/BF03193711 17355033

[c9] Biederman I. (1987). Recognition-by-components: A theory of human image understanding. Psychological Review, 94, 115–147. 10.1037/0033-295X.94.2.115 3575582

[c10] Brunet D., Murray M. M., & Michel C. M. (2011). Spatiotemporal analysis of multichannel EEG: CARTOOL. Computational Intelligence and Neuroscience, 2011, 813870 10.1155/2011/813870 21253358 PMC3022183

[c11] Bülthoff H. H., & Edelman S. (1992). Psychophysical support for a two-dimensional view interpolation theory of object recognition. Proceedings of the National Academy of Sciences, USA of the United States of America, 89, 60–64. 10.1073/pnas.89.1.60 PMC48175 1729718

[c12] Burke D. (2005). Combining disparate views of objects: Viewpoint costs are reduced by stereopsis. Visual Cognition, 12, 705–719. 10.1080/13506280444000463

[c13] Burke D., Taubert J., & Higman T. (2007). Are face representations viewpoint dependent? A stereo advantage for generalizing across different views of faces. Vision Research, 47, 2164–2169. 10.1016/j.visres.2007.04.018 17572467

[c14] Chan M. W., Stevenson A. K., Li Y., & Pizlo Z. (2006). Binocular shape constancy from novel views: The role of a priori constraints. Perception & Psychophysics, 68, 1124–1139. 10.3758/BF03193715 17355037

[c15] Cichy R. M., Khosla A., Pantazis D., Torralba A., & Oliva A. (2016). Comparison of deep neural networks to spatio-temporal cortical dynamics of human visual object recognition reveals hierarchical correspondence. Scientific Reports, 6, 27755 10.1038/srep27755 27282108 PMC4901271

[c16] Cichy R. M., Pantazis D., & Oliva A. (2014). Resolving human object recognition in space and time. Nature Neuroscience, 17, 455–462. 10.1038/nn.3635 24464044 PMC4261693

[c17] Cristino F., Davitt L., Hayward W. G., & Leek E. C. (2015). Stereo disparity facilitates view generalization during shape recognition for solid multipart objects. Quarterly Journal of Experimental Psychology: Human Experimental Psychology, 68, 2419–2436. 10.1080/17470218.2015.1017512 25679983

[c18] Edelman S., & Bülthoff H. H. (1990). Viewpoint specific representation in three-dimensional object recognition (A. I. Memo No. 1239, C. B. I. P Memo No, 53). Retrieved from http://hdl.handle.net/1721.1/6556

[c19] Fabre-Thorpe M. (2011). The characteristics and limits of rapid visual categorization. Frontiers in Psychology, 2, 243 10.3389/fpsyg.2011.00243 22007180 PMC3184650

[c20] Foster D. H., & Gilson S. J. (2002). Recognizing novel three-dimensional objects by summing signals from parts and views. Proceedings Biological Sciences, 269, 1939–1947. 10.1098/rspb.2002.2119 12350257 PMC1691113

[c21] Groppe D. M., Urbach T. P., & Kutas M. (2011). Mass univariate analysis of event-related brain potentials/fields I: A critical tutorial review. Psychophysiology, 48, 1711–1725. 10.1111/j.1469-8986.2011.01273.x 21895683 PMC4060794

[c22] Guthrie D., & Buchwald J. S. (1991). Significance testing of difference potentials. Psychophysiology, 28, 240–244. 10.1111/j.1469-8986.1991.tb00417.x 1946890

[c23] Harris I. M., Dux P. E., Benito C. T., & Leek E. C. (2008). Orientation sensitivity at different stages of object processing: Evidence from repetition priming and naming. PLoS ONE, 3, e2256 10.1371/journal.pone.0002256 18509451 PMC2384001

[c24] Hegdé J. (2008). Time course of visual perception: Coarse-to-fine processing and beyond. Progress in Neurobiology, 84, 405–439. 10.1016/j.pneurobio.2007.09.001 17976895

[c26] Heinze H. J., Hinrichs H., Scholz M., Burchert W., & Mangun G. R. (1998). Neural mechanisms of global and local processing. A combined PET and ERP study. Journal of Cognitive Neuroscience, 10, 485–498. 10.1162/089892998562898 9712678

[c25] Heinze H. J., Johannes S., Munte T. F., & Mangun G. R. (1994). The order of global- and local-level information processing: Electrophysiological evidence for parallel perception processes In Heinz H., Munte T., & Mangun G. R. (Eds.), Cognitive electrophysiology (pp. 102–123). Boston, MA: Birkhauser 10.1007/978-1-4612-0283-7_4

[c27] Hong Liu C., Ward J., & Young A. W. (2006). Transfer between two- and three-dimensional representations of faces. Visual Cognition, 13, 51–64. 10.1080/13506280500143391

[c28] Hummel J. E. (2013). Object recognition In Reisburg D. (Ed.), Oxford handbook of cognitive psychology (pp. 32–46). Oxford, UK: Oxford University Press.

[c29] Hummel J. E., & Stankiewicz B. J. (1996). An architecture for rapid, hierarchical structural description In Inui T. & McCelland J. (Eds.), Attention and performance XVI: On information integration in perception and communication (pp. 93–121). Cambridge, MA: MIT Press.

[c30] Humphrey G. K., & Khan S. C. (1992). Recognizing novel views of three-dimensional objects. Canadian Journal of Psychology/Revue canadien de psychologie expérimentale. 46, 170–190. 10.1037/h0084320 1451040

[c31] Kay K. N., Naselaris T., Prenger R. J., & Gallant J. L. (2008). Identifying natural images from human brain activity. Nature, 452, 352–355. 10.1038/nature06713 18322462 PMC3556484

[c32] Khaligh-Razavi S.-M., & Kriegeskorte N. (2014). Deep supervised, but not unsupervised, models may explain IT cortical representation. PLoS Computational Biology, 10, e1003915 10.1371/journal.pcbi.1003915 25375136 PMC4222664

[c33] Kirchner H., & Thorpe S. J. (2006). Ultra-rapid object detection with saccadic eye movements: Visual processing speed revisited. Vision Research, 46, 1762–1776. 10.1016/j.visres.2005.10.002 16289663

[c34] Koenderink J. J., van Doorn A. J., & Kappers A. M. L. (1992). Surface perception in pictures. Perception & Psychophysics, 52, 487–496. 10.3758/BF03206710 1437481

[c35] Krizhevsky A., Sutskever I., & Hinton G. E. (2012). ImageNet classification with deep convolutional neural networks In Pereira F., Burges C. J. C., Bottou L., & Weinberger K. Q. (Eds.), Advances in neural information processing systems (Vol. 25, pp. 1097–1105). Red Hook, NY: Curran Associates, Inc.

[c36] Lee Y. L., & Saunders J. A. (2011). Stereo improves 3D shape discrimination even when rich monocular shape cues are available. Journal of Vision, 11, 6 10.1167/11.9.6 21849629

[c37] Leek E. C. (1998a). The analysis of orientation-dependent time costs in visual recognition. Perception, 27, 803–816. 10.1068/p270803 10209643

[c38] Leek E. C. (1998b). Effects of stimulus orientation on the identification of common polyoriented objects. Psychonomic Bulletin & Review, 5, 650–658. 10.3758/BF03208841

[c39] Leek E. C., Atherton C. J., & Thierry G. (2007). Computational mechanisms of object constancy for visual recognition revealed by event-related potentials. Vision Research, 47, 706–713. 10.1016/j.visres.2006.10.021 17267003

[c40] Leek E. C., Davitt L. I., & Cristino F. (2015). Implicit encoding of extrinsic object properties in stored representations mediating recognition: Evidence from shadow-specific repetition priming. Vision Research, 108, 49–55. 10.1016/j.visres.2015.01.011 25637853

[c101] Leek E. C., D’Avossa G., Yuen S. L., Hu M., Tainturier M-J., & Rafal R. (2012). Semantic relatedness and visual similarity affect overlapping figures task performance in ventral simultanagnosia: Implications for theories of perceptual integration in human vision. Cognitive Neuropsychology, 29, 569–583. 23521054 10.1080/02643294.2012.752724

[c41] Leek E. C., & Johnston S. J. (2006). A polarity effect in misoriented object recognition: The role of polar features in the computation of orientation-invariant shape representations. Visual Cognition, 13, 573–600. 10.1080/13506280544000048

[c42] Leek E. C., Reppa I., & Arguin M. (2005). The structure of three-dimensional object representations in human vision: Evidence from whole-part matching. Journal of Experimental Psychology: Human Perception and Performance, 31, 668–684. 10.1037/0096-1523.31.4.668 16131241

[c43] Leek E. C., Roberts M. V., Oliver Z. J., Cristino F., & Pegna A. (2016). Early differential sensitivity of evoked-potentials to local and global shape during the perception of three-dimensional objects. Neuropsychologia, 89, 495–509. 10.1016/j.neuropsychologia.2016.07.006 27396674

[c44] Lehmann D., & Skrandies W. (1980). Reference-free identification of components of checkerboard-evoked multichannel potential fields. Electroencephalography and Clinical Neurophysiology, 48, 609–621. 10.1016/0013-4694(80)90419-8 6155251

[c45] Li Y., & Pizlo Z. (2011). Depth cues versus the simplicity principle in 3D shape perception. Topics in Cognitive Science, 3, 667–685. 10.1111/j.1756-8765.2011.01155.x 25164504

[c46] Li Y., Pizlo Z., & Steinman R. M. (2009). A computational model that recovers the 3D shape of an object from a single 2D retinal representation. Vision Research, 49, 979–991. 10.1016/j.visres.2008.05.013 18621410

[c102] Lloyd-Jones T. J., Roberts M. V., Leek E. C., Fouquet N. C., & Truchanowicz E. G. (2012). The Time Course of Activation of Object Shape and Shape+Colour Representations during Memory Retrieval. PLoS ONE, 7(11), e48550 10.1371/journal.pone.0048550 23155393 PMC3498244

[c47] Marr D., & Nishihara H. K. (1978). Representation and recognition of the spatial organization of three-dimensional shapes. Proceedings of the Royal Society of London, Series B: Biological Sciences, 200, 269–294. 10.1098/rspb.1978.0020 24223

[c48] Murray M. M., Brunet D., & Michel C. M. (2008). Topographic ERP analyses: A step-by-step tutorial review. Brain Topography, 20, 249–264. 10.1007/s10548-008-0054-5 18347966

[c49] Navon D. (1977). Forest before trees: The precedence of global feature in visual perception. Cognitive Psychology, 9, 353–383. 10.1016/0010-0285(77)90012-3

[c50] Norman J. F., Swindle J. M., Jennings L. R., Mullins E. M., & Beers A. M. (2009). Stereoscopic shape discrimination is well preserved across changes in object size. Acta Psychologica, 131, 129–135. 10.1016/j.actpsy.2009.03.009 19389660

[c51] Norman J. F., Todd J. T., & Phillips F. (1995). The perception of surface orientation from multiple sources of optical information. Perception & Psychophysics, 57, 629–636. 10.3758/BF03213268 7644323

[c52] Pasqualotto A., & Hayward W. G. (2009). A stereo disadvantage for recognizing rotated familiar objects. Psychonomic Bulletin & Review, 16, 832–838. 10.3758/PBR.16.5.832 19815785

[c53] Pegna A. J., Darque A., Roberts M. V., & Leek E. C. (2017, 5 19). Effects of stereo disparity on early ERP components during classification of three-dimensional objects. The Quarterly Journal of Experimental Psychology, Advance Online Publication 10.1080/17470218.2017.1333129 28524772

[c54] Peyrin C., Baciu M., Segebarth C., & Marendaz C. (2004). Cerebral regions and hemispheric specialization for processing spatial frequencies during natural scene recognition. An event-related fMRI study. NeuroImage, 23, 698–707. 10.1016/j.neuroimage.2004.06.020 15488419

[c55] Peyrin C., Chauvin A., Chokron S., & Marendaz C. (2003). Hemispheric specialization for spatial frequency processing in the analysis of natural scenes. Brain and Cognition, 53, 278–282. 10.1016/S0278-2626(03)00126-X 14607164

[c56] Peyrin C., Michel C. M., Schwartz S., Thut G., Seghier M., Landis T., . . . Vuilleumier P. (2010). The neural substrates and timing of top-down processes during coarse-to-fine categorization of visual scenes: A combined fMRI and ERP study. Journal of Cognitive Neuroscience, 22, 2768–2780. 10.1162/jocn.2010.21424 20044901

[c57] Pizlo Z. (2008). 3D Shape: Its unique place in visual perception. Cambridge, MA: MIT Press.

[c58] Pizlo Z., Sawada T., Li Y., Kropatsch W. G., & Steinman R. M. (2010). New approach to the perception of 3D shape based on veridicality, complexity, symmetry and volume. Vision Research, 50, 1–11. 10.1016/j.visres.2009.09.024 19800910

[c59] Riesenhuber M., & Poggio T. (1999). Hierarchical models of object recognition in cortex. Nature Neuroscience, 2, 1019–1025. 10.1038/14819 10526343

[c60] Rock I., & DiVita J. (1987). A case of viewer-centered object perception. Cognitive Psychology, 19, 280–293. 10.1016/0010-0285(87)90013-2 3581759

[c61] Serre T., Oliva A., & Poggio T. (2007). A feedforward architecture accounts for rapid categorization. Proceedings of the National Academy of Sciences of the United States of America, 104, 6424–6429. 10.1073/pnas.0700622104 17404214 PMC1847457

[c62] Simons D. J., Wang R. F., & Roddenberry D. (2002). Object recognition is mediated by extraretinal information. Perception & Psychophysics, 64, 521–530. 10.3758/BF03194723 12132755

[c63] Tarr M. J., & Bulthoff H. H. (1998). Object recognition in man, monkey, and machine. Cambridge, MA: MIT Press. 10.1016/s0010-0277(98)00026-2 9735534

[c64] Thorpe S., Fize D., & Marlot C. (1996). Speed of processing in the human visual system. Nature, 381, 520–522. 10.1038/381520a0 8632824

[c65] Ullman S. (2007). Object recognition and segmentation by a fragment-based hierarchy. Trends in Cognitive Sciences, 11, 58–64. 10.1016/j.tics.2006.11.009 17188555

[c66] VanRullen R., & Thorpe S. J. (2001). The time course of visual processing: From early perception to decision-making. Journal of Cognitive Neuroscience, 13, 454–461. 10.1162/08989290152001880 11388919

[c67] Welchman A. E., Deubelius A., Conrad V., Bülthoff H. H., & Kourtzi Z. (2005). 3D shape perception from combined depth cues in human visual cortex. Nature Neuroscience, 8, 820–827. 10.1038/nn1461 15864303

[c68] Wexler M., & Ouarti N. (2008). Depth affects where we look. Current Biology, 18, 1872–1876. 10.1016/j.cub.2008.10.059 19062283

[c69] Wismeijer D. A., Erkelens C. J., van Ee R., & Wexler M. (2010). Depth cue combination in spontaneous eye movements. Journal of Vision, 10, 25 10.1167/10.6.25 20884574

